# Elevated H3K27ac in aged skeletal muscle leads to increase in extracellular matrix and fibrogenic conversion of muscle satellite cells

**DOI:** 10.1111/acel.12996

**Published:** 2019-07-20

**Authors:** Jiajian Zhou, Karl K. So, Yuying Li, Yang Li, Jie Yuan, Yingzhe Ding, Fengyuan Chen, Yile Huang, Jin Liu, Wayne Lee, Gang Li, Zhenyu Ju, Hao Sun, Huating Wang

**Affiliations:** ^1^ Department of Chemical Pathology The Chinese University of Hong Kong, Prince of Wales Hospital Shatin, New Territories Hong Kong SAR China; ^2^ Li Ka Shing Institute of Health Sciences, The Chinese University of Hong Kong, Prince of Wales Hospital Shatin, New Territories Hong Kong SAR China; ^3^ Department of Orthopaedics and Traumatology The Chinese University of Hong Kong, Prince of Wales Hospital Shatin, New Territories Hong Kong SAR China; ^4^ Key Laboratory of Regenerative Medicine of Ministry of Education Institute of Aging and Regenerative Medicine Jinan University Guangzhou China

**Keywords:** aging, enhancer, extracellular matrix, H3K27ac, JQ1, satellite cell, skeletal muscle

## Abstract

Epigenetic alterations occur in various cells and tissues during aging, but it is not known if such alterations are also associated with aging in skeletal muscle. Here, we examined the changes of a panel of histone modifications and found H3K27ac (an active enhancer mark) is markedly increased in aged human skeletal muscle tissues. Further analyses uncovered that the H3K27ac increase and enhancer activation are associated with the up‐regulation of extracellular matrix (ECM) genes; this may result in alteration of the niche environment for skeletal muscle stem cells, also called satellite cells (SCs), which causes decreased myogenic potential and fibrogenic conversion of SCs. In mice, treatment of aging muscles with JQ1, an inhibitor of enhancer activation, inhibited the ECM up‐regulation and fibrogenic conversion of SCs and restored their myogenic differentiation potential. Altogether, our findings not only uncovered a novel aspect of skeletal muscle aging that is associated with enhancer remodeling but also implicated JQ1 as a potential treatment approach for restoring SC function in aging muscle.

## 1 INTRODUCTION

Skeletal muscle is a contractile, postmitotic tissue composed of multinucleated muscle cells known as myofibers, which are formed by the fusion of differentiated mononuclear muscle cells. Skeletal muscle accounts for 30%–50% of body mass in humans and exhibits an extremely low turnover in the absence of disease or injury (Almada & Wagers, 2016). Meanwhile, muscle possesses remarkable regenerative capacity mediated by adult muscle stem cells, also called satellite cells (SCs) that reside in close association with individual myofibers, underneath the fiber's basal lamina (Mauro, 1961; Yin, Price, & Rudnicki, 2013). Responding to muscle injury, SCs will be activated and proliferate, differentiate, and fuse to existing damaged fibers or fuse with one another to form myofibers de novo. Meanwhile, a subpopulation of SCs returns to quiescence to replenish the stem cell pool (Yin et al., 2013). As we age, the mass, function, and regenerating capacity of muscle gradually decline, affecting mobility, voluntary function, and quality of life (Almada & Wagers, 2016; Bengal, Perdiguero, Serrano, & Muñoz‐Cánoves, 2017). For example, ~1% of skeletal muscle mass and ~2.5%–4% of strength in lower limb muscle is lost per year starting 30th life‐year (Goodpaster et al., 2006; Keller & Engelhardt, 2013). It is thus imperative to investigate mechanisms accounting for the age‐related muscle loss and functional decline. Changes at all levels, including gene expression, histone modification, DNA methylation, and physical changes in muscle stem cell environment, or niche, have been found to be associated with aging (Liu et al., 2013; Sahu et al., 2018; Stearns‐Reider et al., 2017; Su et al., 2015; Vinel et al., 2018; Zykovich et al., 2014). For instance, one of the studies of gene expression in aging muscle revealed that mitochondrial dysfunction is a major age‐related phenomenon and highlighted the beneficial effects of maintaining a high physical capacity in the prevention of age‐related muscle function decline (Su et al., 2015). In another report, transcriptome‐wide analysis demonstrated that the expression of extracellular matrix (ECM) genes is up‐regulated during muscle aging (Zahn et al., 2006). The myofiber basal lamina is comprised of an ECM network that is in direct contact with SCs and ECM plays an essential role in maintaining microenvironmental homeostasis and SC function; the increased ECM levels in aging niche thus lead to deregulated behaviors of SCs. Specifically, SCs displayed decreased myogenic potential but increased expression of fibrogenic genes in aged muscle (Croisier, 2004; Gargioli, Coletta, De Grandis, Cannata, & Cossu, 2008; Stearns‐Reider et al., 2017). This study highlights the impact of niche alterations on the SC function during aging. Still, it is yet to investigate the causes of ECM increase during muscle aging.

Alterations at epigenetic levels are also known to be associated with aging (López‐Otín, Blasco, Partridge, Serrano, & Kroemer, 2013; Sen, Shah, Nativio, & Berger, 2016). For example, Zykovich et al. (2014) uncovered that DNA hypermethylation at gene regions is associated with aging muscle. However, potential alterations of histone marks in aging muscle have not been investigated. For example, H3K27ac enrichment is a hallmark of enhancers and is often used to define typical enhancers (TEs) and super‐enhancers (SEs) with the latter bear unusually high levels of H3K27ac and binding of key transcriptional regulators (Creyghton et al., 2010; Hnisz et al., 2013; Whyte et al., 2013). Several studies have shown the deregulation of H3K27ac and enhancers in aging tissues/cell types (Benayoun et al., 2018; Cheung et al., 2018; Moskowitz et al., 2017; Ucar et al., 2017). For instance, Benayoun et al. found that increased H3K27ac and H3K4me3 levels lead to the up‐regulation of interferon‐related pathways in heart, liver, cerebellum, olfactory bulb, and primary cultures of neural stem cells (NSCs) throughout mouse lifespan (Benayoun et al., 2018); Moskowitz et al. (2017) reported an increased enhancer openness, while a loss of promoter openness in aging CD8^+^ T cells. Our recent report also showed that SEs display substantial enhancer remodeling during myoblast differentiation (Peng et al., 2017) but it is not known if enhancer alterations are associated with muscle aging.

In this study, we examined the changes of a panel of histone marks and found H3K27ac is markedly increased during aging in human skeletal muscle tissues. We next identified aging‐related enhancer alterations and found them associated with the up‐regulation of ECM genes. Furthermore, comparison of transcriptomes in young and aged SCs demonstrated that an age‐related fibrogenic conversion of SCs. In mice, treatment of aging muscles with JQ1, an inhibitor of enhancer activation, reverted the ECM up‐regulation and fibrogenic conversion of SCs, thus restored myogenic potential of SCs, suggesting that ECM increase in aging muscle is a result of enhancer activation and JQ1 can be a potential treatment approach for restoring SC function in aging muscle.

## 2 RESULTS

### 2.1 Increase in H3K27ac mark in aged human muscle

**Figure 1:**
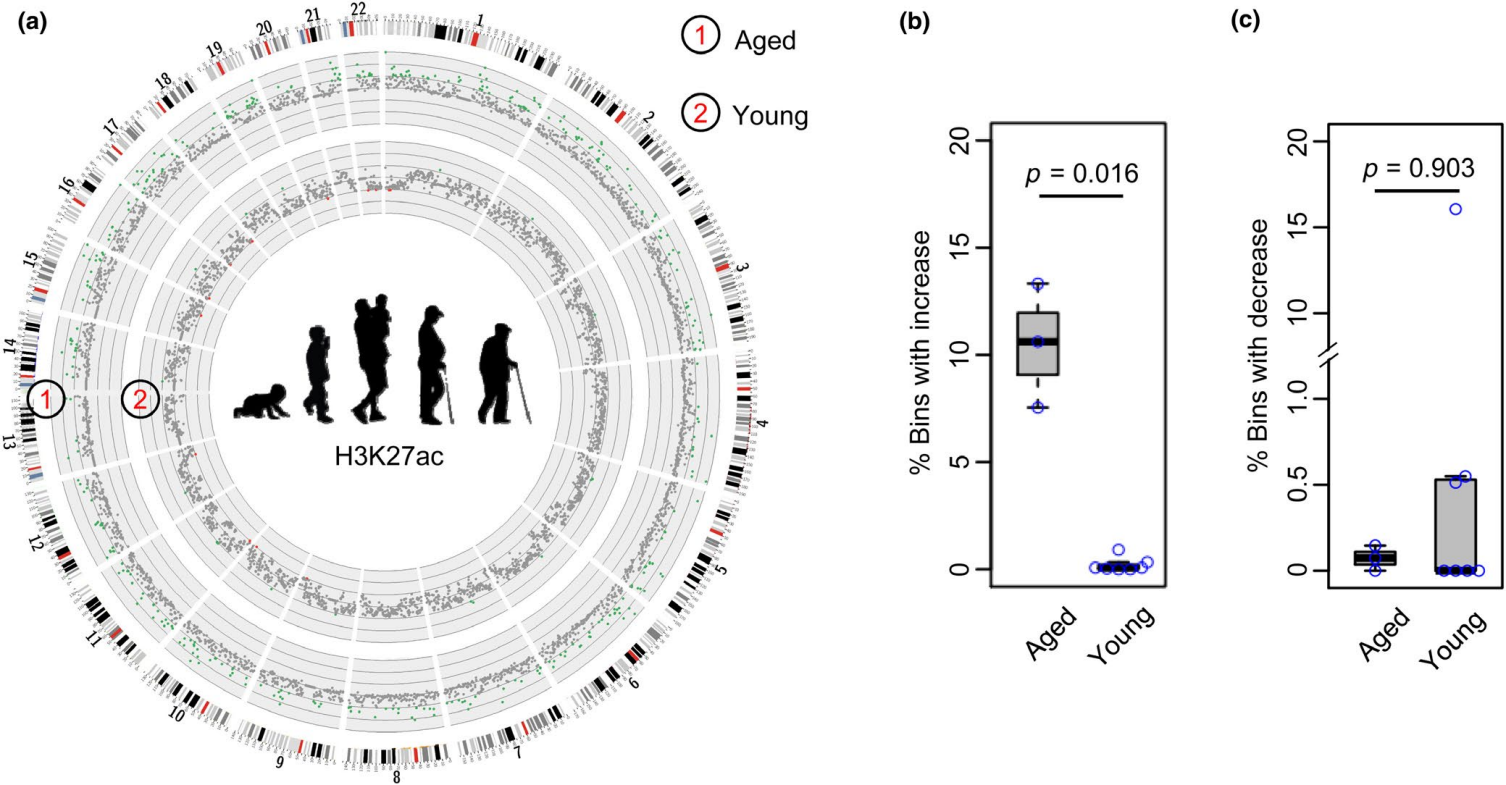
Genome‐wide increase in H3K27ac mark in aged human muscle. (a) Circos plot illustrating the increase in H3K27ac in aged versus young muscles. The green dot represents a bin with increased H3K27ac (*z*‐score > 2), while the red dot represents a bin with decreased H3K27ac (*z*‐score < −2). (b and c) Boxplot showing that the percentage of bins with increased but not decreased H3K27ac is significantly higher in the aged versus young group

Recent studies showed that age‐associated transcriptional alterations during muscle aging are strongly associated with metabolic pathways and DNA methylation (Su et al., 2015; Zykovich et al., 2014), but the changes on histone modifications remain unexplored. To fill the gap, we collected available ChIP‐seq datasets for H3K4me1, H3K4me3, H3K27me3, H3K9me3, and H3K27ac in human muscles (annotated as gastrocnemius medialis and psoas muscles) at various ages (age range: 3–72 years, see Experimental procedures). Due to the limited number of samples, the datasets were coarsely classified into two groups: (a) young from ≤50 years; and (b) aged from >50 years (Table S1). To detect any difference, we divided the reference genome (hg19) into 1 Mb bins and quantified the difference of each mark in binned regions between young and aged group as z‐score, which is the number of standard deviations (*SD*s) away from the mean of normalized read counts of young muscle samples. By comparing all binned regions across the genome, the circos plot demonstrated that the level of H3K27ac mark is evidently increased in aged versus young group (Figure 1a). Moreover, the percentage of bins with increased H3K27ac in the aged group is significantly higher compared with young group (Figure 1b) while the bins with decreased H3K27ac is unchanged between the two groups (Figure 1c). When the comparison was performed on other histone marks including H3K4me1, H3K4me3, H3K9me3, and H3K27me3, no significant differences were detected in aged versus young group (Figure S1). Together, the above results indicate that the increase in H3K27ac is a hallmark of aged human skeletal muscle tissue, warranting further investigation of its implication.

### 2.2 Identification of age‐related enhancer alterations during human muscle aging

**Figure 2:**
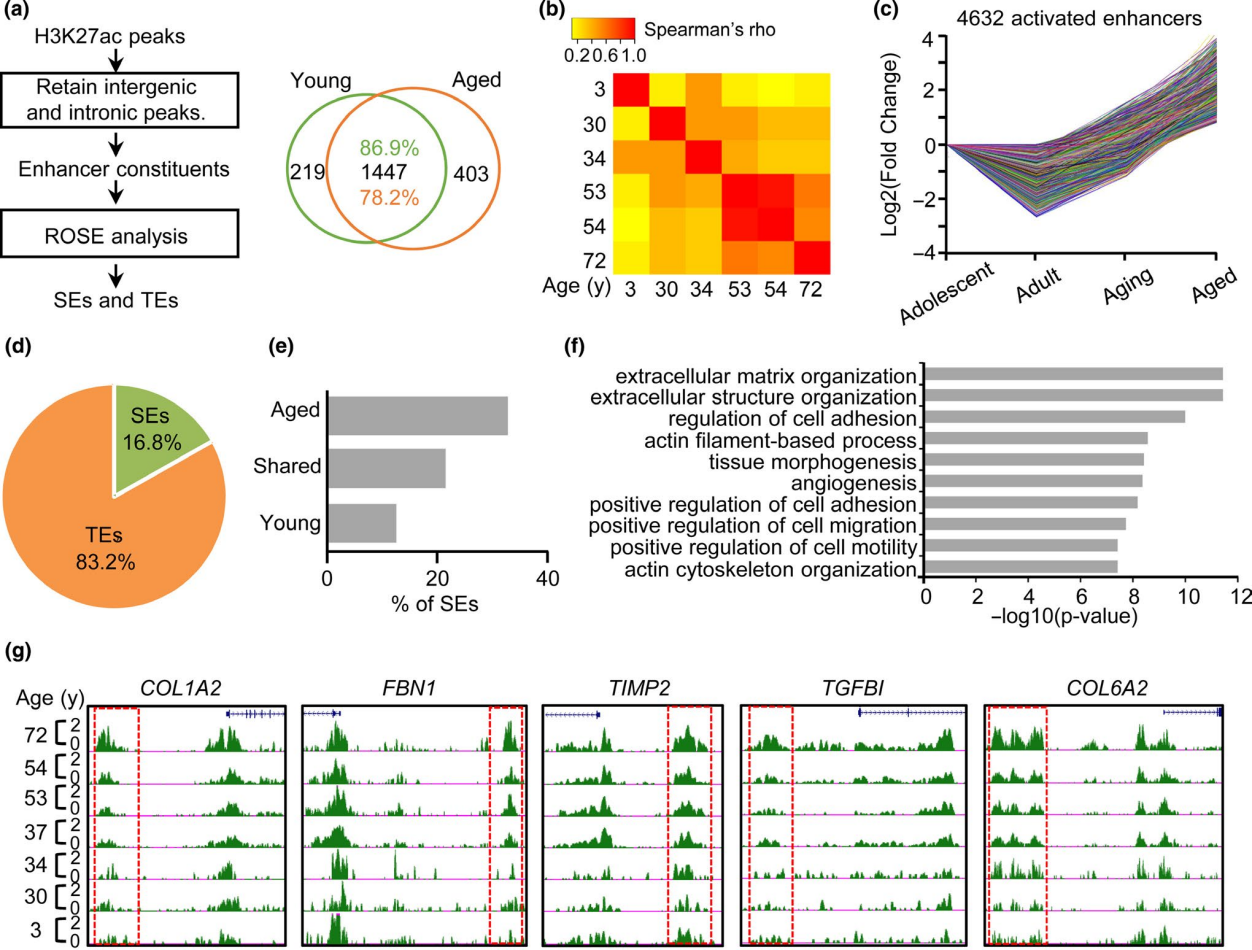
Identification of age‐related enhancer alterations during human muscle aging. (a) Illustration of the computational pipeline for identifying typical enhancers (TEs) and super‐enhancers (SEs). (b) Venn diagram showing the changed profiles of SEs between young and aged groups. (c) Heatmap showing Spearman's correlation coefficient (Spearman's *ρ*) of SEs signals among different age samples. (d) Line plot showing the fold change (log2) of enhancer constituents with increased signal during muscle aging. The enhancer cluster is identified through STEM analysis. (e) The distribution of the above activated enhancers in TEs or SEs. (f) Bar plot showing a large proportion of aged specific SEs contain at least one activated enhancer. (g) Gene ontology (GO) analysis shows that the proximal genes of the above activated enhancers are enriched in extracellular matrix (ECM) terms. (h) Snapshots of H3K27ac signals in cognate enhancers of five ECM genes

As H3K27ac delineates active enhancers, and its signal strength is positively correlated with enhancer activity (Creyghton et al., 2010; Li, Notani, & Rosenfeld, 2016; Peng et al., 2017), we speculated that the increase in H3K27ac might alter the enhancer landscape during muscle aging. To test this notion, we defined TEs and SEs according to the H3K27ac signals (Peng et al., 2017; Figure 2a, Experimental procedures) and examined age‐related enhancer alterations. An average of 900–1,600 SEs and 22,340–23,072 TEs were defined in each sample; a total number of 22,518 TEs and 1,666 SEs were defined in the young group while 22,675 TEs and 1,810 SEs in the aged group (Figure 2b, Table S2). Interestingly, a large proportion (>70%) of SEs is shared between the young and aged groups, while 403 SEs are specific to aged muscles (Figure 2b). When examining the correlation of H3K27ac signals of SEs in all samples using a heatmap analysis (see Experimental procedures), progressive changes in SE profiles were readily observed during muscle aging; a high correlation was observed between the two consecutive ages but gradually decreases as the age increases (Figure 2c). To further explore the dynamics of enhancer remodeling during muscle aging, we categorized all muscle samples into four groups: adolescent, adult, aging, and aged (adolescent: 3–23 years, three samples; adult: 30–40 years, two samples; aging: 50–60 years, two samples; aged: >70 years, one sample; see Experimental procedures). All enhancer constituents from all samples were merged to form a uniform enhancer profile, and RPKM value for each enhancer constituent in each sample was then calculated. Clustering analysis for all peaks based on their RPKM values was then performed using Short Time‐series Expression Miner (STEM; Ernst & Bar‐Joseph, 2006). By clustering ~15,000 enhancer constituents with at least twofold changes of RPKM values between adolescent and aged group, 4,632 comprised a cluster with a gradual increase in H3K27ac signal during aging (Figure 2d), while only a very small number of enhancer clusters (<100 enhancers) displayed a decreased H3K27ac signal (Figure S2). Altogether, our results confirmed a global increase in H3K27ac level during muscle aging. Interestingly, the majority (3,854, 83.2%) of the activated enhancers are from TEs and 16.8% (778) from SEs (Figure 2e). Still, we observed more than 30% of aged specific SEs contain at least one activated enhancer constitute, which is higher than those shared or young‐specific SEs (Figure 2f), suggesting that SE activation is a hallmark of muscle aging. Furthermore, GO enrichment analysis showed that the adjacent genes associated with activated enhancers are markedly enriched for extracellular matrix (ECM) structure and organization (Figure 2g), suggesting a possible role for the enhancer activation in upregulating ECM gene expression during aging. Expectedly, an increase in H3K27ac signal was observed in the cognate enhancers of ECM genes, such as *COL1A2*, *FBN1*, *TIMP2*, *TGFBI,* and *COL6A2* (Figure 2h). Collectively, our results revealed that remodeling of enhancer landscape is prevalent during muscle aging and enhancer activation may be associated with ECM gene up‐regulation.

### 2.3 Enhancer alterations are associated with up‐regulation of ECM genes in aged human muscle

**Figure 3:**
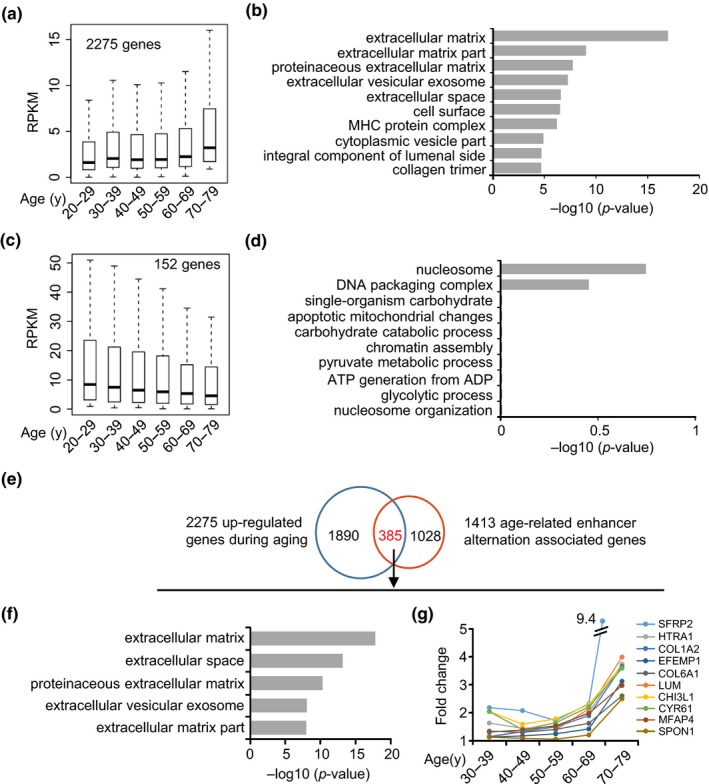
Enhancer alterations are associated with up‐regulation of extracellular matrix (ECM) genes in aged human muscle. (a) Boxplot showing a group of genes (2,275 genes) with a gradual increase in expression during muscle aging and (b) GO enrichment analysis of those genes. (c) Boxplot showing a group of genes (152 genes) with a gradual decrease in expression during muscle aging and (d) GO enrichment analysis of those genes. (e) The overlapping analysis of up‐regulated genes and activated enhancer‐associated genes. (f) GO analysis of the overlapped genes (385) revealed enriched ECM‐related terms. (g) Line plot showing the expression of the top 10 most increased ECM genes during muscle aging

To further test whether the above discovered enhancer activation leads to ECM gene up‐regulation, we obtained RNA‐seq profiles of 454 human muscle tissues from GTEx (The GTEx Consortium, 2015). All samples were categorized into six age groups: 20–29, 30–39, 40–49, 50–59, 60–69, and 70–79 years (Table S1, see Experimental procedures). STEM analysis was carried out to examine the gene expression dynamics. As shown in Figure 3a, a group of genes (2,275 genes) with a gradual increase in expression during aging and a relatively small group (152 genes) with a decrease in expression (Figure 3c) were uncovered (Table S3). Expectedly, GO enrichment analysis showed that the up‐regulated genes are indeed strongly enriched with ECM terms (Figure 3b, Table S3), while the down‐regulated genes are marginally associated with nucleosome and DNA packaging complex (Figure 3d, Table S3). We further overlapped the activated enhancers with the increased genes and found that 385 (27%) out of 1,413 genes associated with activated enhancers are up‐regulated (Figure 3e, Table S3) including *SFRP2, HTRA1, COL1A2, EFEMP1, and COL6A1* (Figure 3g, Figure S3), which is in line with the activating role of enhancers. GO enrichment analysis again demonstrated that these genes are highly enriched for ECM terms (Figure 3f), suggesting that ECM up‐regulation is tightly associated with activated enhancers.

### 2.4 Enhancer activation in aging muscle drives ECM gene up‐regulation and fibrogenic conversion of SCs in mice

**Figure 4:**
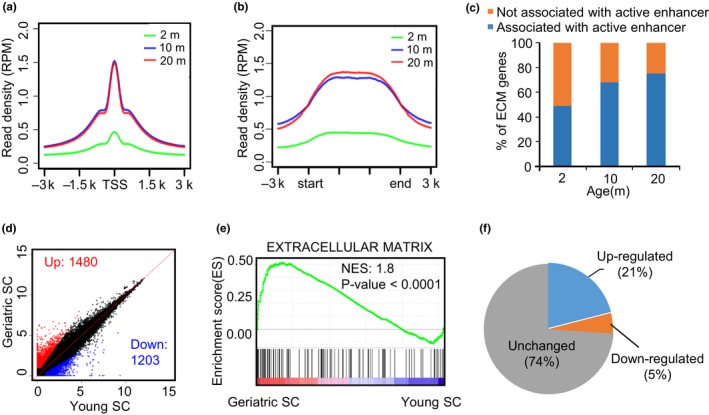
Enhancer activation in aging muscle drives extracellular matrix (ECM) gene up‐regulation and fibrogenic conversion of satellite cells (SCs) in mice. (a) Density plot showing the level of H3K27ac signals around TSSs of all expressed genes. (b) Density plot showing the level of H3K27ac around cognate enhancers associated with ECM genes. (c) Bar plot showing the percentage of ECM genes regulated by at least one activated enhancer. In 2‐, 10‐ and 20‐month‐old muscles, ECM genes were divided into two groups: “associated with active enhancer,” which means that the adjacent enhancer of a particular ECM gene is marked by H3K27ac; “not associated with active enhancer” means that the adjacent enhancer of a particular ECM gene is not marked by H3K27ac. (d) Scatter plot showing the differentially expressed genes in geriatric versus young SCs. (e) GSEA analysis shows that the above up‐regulated genes in geriatric SCs are enriched in ECM. (f) Pie chart showing ~20% of ECM genes are up‐regulated in geriatric versus young SCs

To further test the notion that ECM gene increase is caused by enhancer activation, we resorted to the mouse muscle system. We first carried out H3K27ac ChIP‐seq using tibialis anterior (TA) muscle tissues from 2‐, 10‐, and 20‐month‐old mice. Signs of aging including appearance of centrally localized nuclei and degenerated myofibers indeed were evident in 20‐month‐old mouse muscles compared with 2‐month‐old ones (Figure S4a; Lin et al., 2018). Consistent with the findings in human, an increased H3K27ac signal was detected during muscle aging (Figure 4a). Nevertheless, the total protein level of H3K27ac detected by Western blot did not appear to increase during muscle aging (Figure S4b,c). We reason that the increased H3K27ac signal is from ~10%–15% genome regions (Figure 1b), which may not correlate with the total H3K27ac protein level in cells. Interestingly, the level of H3K27ac is highly elevated as early as 10 months and remained high in 20 months, suggesting that the enhancer remolding possibly starts before the actual aging signs occur. To validate whether the increased enhancer signal is associated with ECM gene as seen in human samples, we found a significantly higher level of H3K27ac in the ECM‐associated enhancers (Figure 4b); meanwhile, a higher portion of ECM genes are associated with active enhancers in old (10 and 20 months) versus young (2 months) muscles (Figure 4c, Table S4), indicating that the increase in H3K27ac in enhancers is associated with the up‐regulation of ECM genes during mouse muscle aging.

Since ECM over‐accumulation in aging muscle results in an increase in stiffness of niche environment thus leading to a fibrogenic conversion of SCs (Stearns‐Reider et al., 2017), we next examined whether the observed ECM up‐regulation in aged muscle leads to such deregulation of SCs. By analyzing microarray data from SCs isolated from geriatric and young mice (Sousa‐Victor et al., 2014), 1,480 genes were found to be up‐regulated in geriatric SCs and 1,203 genes down‐regulated (Figure 4d, Table S4). GSEA enrichment analysis further showed that the up‐regulated genes are indeed enriched in ECM (Figure 4e), suggesting a fibrogenic conversion may occur in aged SCs. Among the 124 ECM component genes, 21% are up‐regulated, while only 5% are down‐regulated (Figure 4f). Taken together, our analyses show that ECM up‐regulation in mouse muscle tissue might change the niche environment and result in a fibrogenic conversion of SCs.

### 2.5 JQ1 treatment reverts the expression of some ECM genes in aged muscle

**Figure 5:**
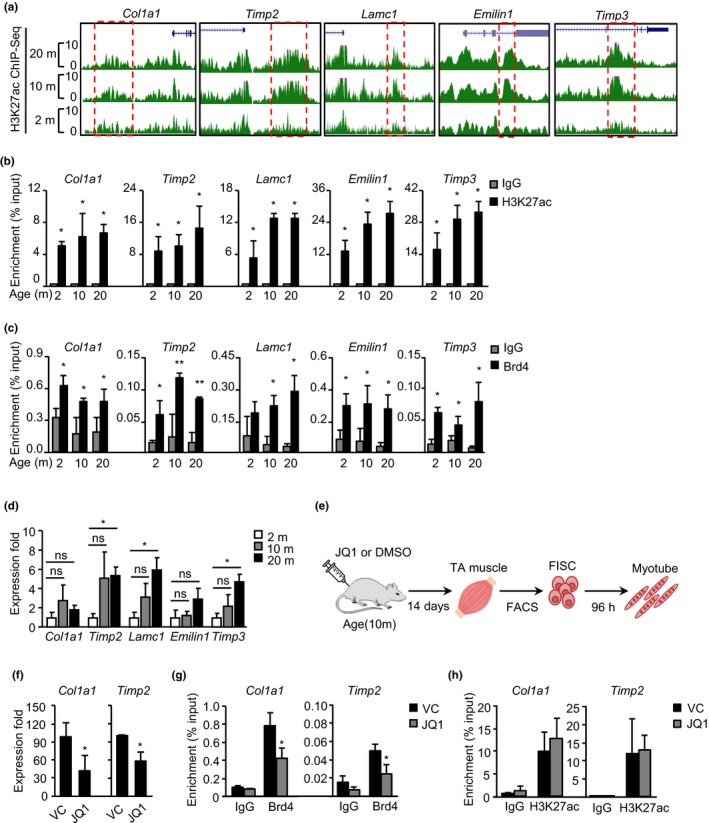
JQ1 treatment reverts the expression of some extracellular matrix (ECM) genes in aged muscle. (a) Snapshots of H3K27ac binding profiles on five selected ECM enhancers in 2‐, 10‐, and 20‐month‐old muscles. (b) Binding of H3K27ac and Brd4 (c) on selected ECM enhancers in the above muscles was measured by ChIP‐PCR. (d) RNA expression levels of the above selected ECM genes were quantified by RT‐qPCR. (e) Schematic overview of intraperitoneal injection of JQ1 and experimental design. (f) Down‐regulation of ECM genes (*Col1a1, Timp2*) in skeletal muscle treated with JQ1. (g) Brd4 or H3K27ac (h) binding on the above two genes was measured by ChIP‐PCR. The p‐value was determined by Student's *t* test: *p < 0.05, **p < 0.01, ***p < 0.001, ns, not significant

JQ1 is a drug that inhibits global enhancer activities through targeting the function of the bromodomain and extra‐terminal domain (BET) proteins in enhancer regions. Recent studies showed that JQ1 not only challenges progression of several forms of cancer (Asangani et al., 2014; Delmore et al., 2011; Roe, Mercan, Rivera, Pappin, & Vakoc, 2015; Zuber et al., 2011), but also counteracts cancer cachexia in tumor‐bearing mice through preventing muscle and adipose tissue wasting (Segatto et al., 2017). Therefore, we asked whether JQ1 treatment could delay the process of muscle aging through inhibiting the increased ECM enhancer activities in aged mouse muscle. Five selected ECM genes, including *Col1a1*, *Timp2*, *Lamc1*, *Emilin1*, and *Timp3*, displayed evident increase in H3K27ac signal in old (10‐ or 20‐month‐old) versus young (2‐ or 4‐month‐old) muscles by both ChIP‐seq (Figure 5a) and ChIP‐PCR (Figure 5b and Figure S4d). Persistent binding of Brd4 (a BET component) was also detected (Figure 5c), suggesting these enhancers are indeed activated by Brd4. Concomitantly, their expressions showed an overall increase in trend during the aging course and the increase was significant comparing in 20‐ versus 2‐month‐old muscles (Figure 5d). Moreover, knockdown of Brd4 in 10T1/2 fibroblast cells down‐regulated their expression levels (Figure S4e,f). The above results justified the use of JQ1 to inhibit ECM gene activation. We thus performed intraperitoneal injection of JQ1 at 50mg/kg daily on 10‐month‐old mouse for 14 days (see Experimental procedures, Figure 5e). As a result, the expression of some ECM genes, for example *Col1a1* and *Timp2,* was significantly down‐regulated demonstrating that JQ1 treatment could revert the expression of some ECM genes in aged muscle (Figure 5f and Figure S4g–h). As expected, Brd4 binding on the loci was also found to be decreased (Figure 5g); H3K27ac binding, on the other hand, was not changed (Figure 5h). This is consistent with findings from Lee et al. (2017) showing Brd4 loss reduces gene induction through preventing Pol II binding but has no significant impact on H3K27ac enrichment on enhancers. In addition, the JQ1 administration did not appear to cause obvious toxic effect in mice. Instead, we found JQ1 treatment appears to slightly increase the lean mass percentage in aging mouse by 7.5% (data not shown), suggesting that JQ1 can potentially ameliorate the obesity condition associated with sarcopenia.

### 2.6 JQ1 treatment inhibits fibrogenesis and restores myogenic ability in aging SC

**Figure 6:**
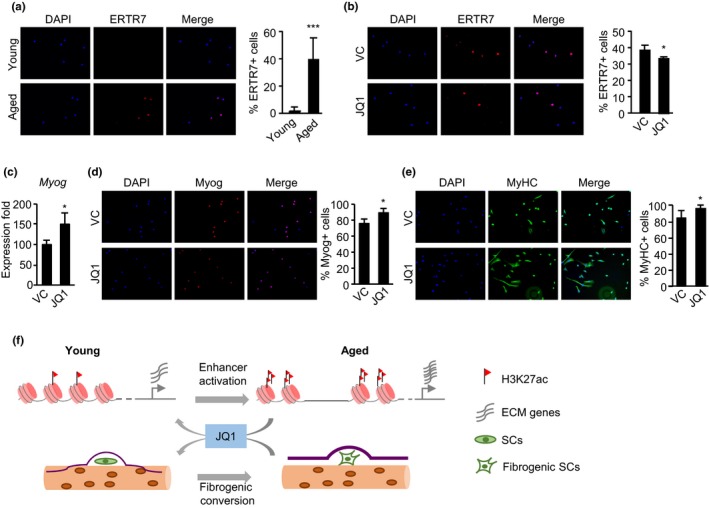
JQ1 treatment inhibits fibrogenesis and restores myogenic ability in aging SC. (a) Staining of ERTR7 in freshly isolated satellite cells (SCs) (FISCs) from 2‐ or 10‐month‐old muscles; quantification of the percentage of positively stained cells is shown on the right. (b) JQ1 treatment decreased the percentage of ERTR7 positive FISCs in 10‐month‐old muscles. (c) The above isolated SCs were differentiated for 96 hr, and the mRNA expression of Myogenin gene was examined. (d) The above differentiated cells were stained for Myogenin, and the positively stained cells were quantified. (e) The above differentiated cells were stained for MyHC, and the positively stained cells were quantified. (f) Schematic mechanistic model of skeletal muscle aging. H3K27ac is markedly increased in human or mouse muscle during aging, which leads to the enhancer activation and subsequent up‐regulation of extracellular matrix (ECM) genes. It causes the alteration of SC niche and fibrogenic conversion of SCs. In mice, treatment of aging muscles with JQ1, an inhibitor of enhancer activation, reverted the ECM up‐regulation and fibrogenic conversion of SCs

Previously, Stearns‐Reider et al. (2017) have demonstrated the occurrence of fibrogenesis and hampered myogenic ability in SCs caused by alterations in aged niche environment. To examine whether JQ1 treatment could prevent the fibrogenic shift of SCs, we FACS sorted SCs of high purity (Figure S5) and stained with ERTR7, a known fibrogenic marker (Brack et al., 2007; Lepper, Conway, & Fan, 2009; Stearns‐Reider et al., 2017). Our results revealed 40% of FISCs from aged muscle (Figure 6a) and 13% in cultured SCs (data not shown) were ERTR7^+^ while 2% positive staining (Figure 6a) on young muscle, which is consistent with the prior report (Brack et al., 2007; Stearns‐Reider et al., 2017). Meanwhile, JQ1 treatment led to a significant decrease (12%) of the percentage of positively stained cells (Figure 6b). Moreover, when assessing whether the myogenic potential of the SCs was restored by JQ1 treatment, we found that the mRNA expression of the early myogenic marker, Myogenin (*Myog*), was significantly up‐regulated by JQ1 treatment when the SCs were differentiated (Figure 6c); consistently, immunofluorescence (IF) staining for Myogenin or a late myotube marker, myosin heavy chain (MyHC) showed an increased percentage of positively stained cells in JQ1‐treated cells (Figure 6d,e). Collectively, our results highlight that JQ1 treatment of aging muscle can to some extent revert SC fibrogenesis and SC myogenic potential.

## 3 DISCUSSION

In this study, we reported the first epigenomic survey in aging human muscle and the remodeling of enhancer profiles during muscle aging. The results demonstrated that global gain of H3K27ac but not other histone modification levels is a hallmark in the aging muscle from both human and mouse. As a result, enhancer activation is prevalent and associated with up‐regulation of ECM genes, which may lead to alterations of SC niche and fibrogenic conversion of SCs. Treatment of JQ1 in aging muscles inhibited enhancer activation and restored ECM gene expression, thus led to reduced fibrogenesis and improved the myogenic potential of SCs (Figure 6f). Altogether, our findings have provided an explanation for previously discovered increased ECM expression in aging muscle tissues and identify BET blockade as a potential approach to delay the progression of muscle aging through modulating enhancer activity.

Aging is accompanied by a decline in skeletal muscle mass and stem cell function (Goodell & Rando, 2015; Liu & Rando, 2011). In human muscle, we for the first time found that H3K27ac mark is gradually increased during aging through integrative analyses of a panel of histone ChIP‐seq datasets. We speculated that increased H3K27ac might promote the expression of crucial regulators associated with the age‐related decline of muscle function. Indeed, through integrative analyses of RNA‐seq and H3K27ac profiles, we further demonstrated that the activated enhancers are associated with ECM genes which are up‐regulated in aged muscle, thus expanding the previous finding showing age‐related ECM alterations in aging muscle (Brack et al., 2007; Stearns‐Reider et al., 2017). Nevertheless, we must point out that our analyses were performed using bulk muscle tissues which compromise of many different cell types, including muscle fibers, endothelial cells, fibro‐adipogenic progenitors (FAPs), and fibroblasts, satellite cells. Therefore, the source of H3K27ac increase and ECM activation during muscle aging remains unclear. The increase in H3K27ac binding is very likely to be derived from resident muscle fibroblasts as these cells are considered as the major contributor to the ECM of skeletal muscle (Thomas, Engler, & Meyer, 2015). Moreover, Stearns‐Reider et al. (2017) demonstrated that aging drives ECM gene expression changes in fibroblasts; however, a general increase was not observed. For example, Col3a1 was decreased while Col4a3 were up‐regulated in aged fibroblasts, suggesting there may be other contributors to the overly increased ECM gene expressions in our study. The future employment of single‐cell sequencing technologies may allow us to investigate the gene expression changes at single‐cell level and bridge to a panorama view of skeletal muscle aging, including dynamics of cell populations, the transcriptome, and epigenome dynamics of each cell type. It is also worth to point out that different types of skeletal muscles may possess different susceptibility to aging process. For example, it was found that in elderly women the greatest rates of age‐related loss of skeletal muscle mass in the lower limbs showed in the psoas major, while the smallest loss showed in soleus muscle (Ikezoe, Mori, Nakamura, & Ichihashi, 2011). In the current study, H3K27ac ChIP‐seq obtained from ENCODE was mainly performed using gastrocnemius medialis and psoas muscles while tibialis anterior muscles were used by us for majority of mouse experiments. In the future, it will be interestingly to explore whether the enhancer activation of ECM genes occurs in all muscles.

Our further analyses also confirmed the phenomenon in aging mouse tissue and further demonstrated that age‐related ECM up‐regulation would cause niche alterations and consequently influence SCs function. This is in line with the notion that maintenance of epigenome integrity and muscle SCs niche is a crucial element to prevent the age‐related decline of skeletal muscle function (Bengal et al., 2017; Liu et al., 2013; Segatto et al., 2017; Zykovich et al., 2014). Thus, development of the treatment to prevent epigenetic alternation and maintain muscle SC niche should be an important goal to delay the progression of muscle aging and improve the life quality of the elderly. To this end, our results highlight a new therapeutic direction to manage and prevent the age‐related decline of muscle functions by inhibiting the gradual increase in enhancer activities, thus slowing down the aging process of skeletal muscle. JQ1 derivatives are a new class of drugs in cancer therapy now under investigation in human clinical trials (Berthon et al., 2016; von Schaper, 2016). Recent studies also have demonstrated that BET inhibitors exhibit profound anti‐fibrotic effects in rodent models of organ failure (Ding et al., 2015; Duan et al., 2017; Stratton et al., 2017). These studies build on the mechanistic rationale that BET inhibitors inhibit the binding of BRD4, which is a nodal, positive regulator of pro‐fibrotic gene expression, and promote extracellular matrix (ECM) deposition and tissue remodeling (Stratton et al., 2017). In this report, our results highlight that BET inhibitors may be beneficial to aged muscle owing to their ability to restrain enhancer activities of ECM genes. Interestingly, a very recent study also showed that JQ1 could counteract muscle wasting condition known as cancer cachexia in tumor‐bearing mice (Segatto et al., 2017). In the scenario of muscle aging, we presented that JQ1 treatment reverts the expression of ECM genes to their levels in the young state, which improves the niche environment of SCs. Indeed, we observed decreased ERTR7^+^ staining in freshly isolated SCs (FISCs) and up‐regulation of Myogenin gene (*Myog*) and Myogenin or MyHC staining when the cells were differentiated, indicating that JQ1 treatment can possibly prevent the fibrogenic shift and restore the myogenic ability of SCs in aged muscle. Nevertheless, the long‐term effects of JQ1 treatment still need to be carefully evaluated to acquire solid evidence of its usage on ameliorating muscle aging conditions.

## 4 EXPERIMENTAL PROCEDURES

### 4.1 Data source

H3K27ac, H3K27me3, H3K4me3, H3K4me1, and H3K9me3 histone ChIP‐seq profiles were downloaded from Epigenome Roadmap project, ENCODE project (https://www.encodeproject.org/) and IHEC project (http://epigenomesportal.ca/ihec/) with the annotation of gastrocnemius medialis, psoas muscle, and skeletal muscle. The expression level (Reads Per Kilobase Million, RPKM) for each gene in 454 muscle tissues was downloaded from GTEx portal (V6 release, https://www.gtexportal.org/home/datasets, login required), which were calculated from RNA‐seq datasets with “Skeletal Muscle” annotation in metadata; microarray datasets in skeletal muscle satellite cells (SCs) were obtained from GEO (GSE53724; Sousa‐Victor et al., 2014); H3K27ac ChIP‐seq datasets in skeletal muscle tissues from 2‐, 10‐, and 20‐month‐old mice were generated in this study and deposited in GSE122867. All datasets used in this study were described in Table S1.

### 4.2 ChIP‐seq analysis

All histone ChIP‐seq datasets from public databases were subjected to different downstream analyses, depending on the availability of data formats. Histone signals in bigwig format obtained from IHEC were normalized to the corresponding input data by deepTools2 (Ramírez et al., 2016). Raw reads from ENCODE and Epigenome roadmap projects were mapped to the human genome (hg19) by bowtie2; for datasets without raw reads, the alignment coordinates were converted to bam format using bedtools and samtools. Peaks were called by MACS2 with default parameters. Samples with a low amount of reads or a low ChIP enrichment efficiency were eliminated for further analyses. All alignment results were then converted to coverage bigwig files and normalized to the corresponding input using deepTools2 (Ramírez et al., 2016). To build a universal H3K27ac peak atlas of muscle tissues, the peaks of all muscle tissues were concatenated and overlapping peaks in at least two samples were merged; the merged peaks and the set of nonoverlapping peaks were combined to the final peak atlas. The same scheme was applied to generate the peak atlas of other histone modifications. A *z*‐score, which is the number of *SD*s away from the mean normalized read count of the young group, was used to quantitatively measure the difference of histone modifications at a specific region during muscle aging; a specific region with z‐score below −2 or above +2 was considered as showing significant gain or loss of a particular histone modification, respectively.

### 4.3 Gene expression analysis

A total of 454 gene expression profiles in human skeletal muscle samples were obtained from the GTEx project (The GTEx Consortium, 2015). The expression profiles were then roughly categorized into six groups with different age ranges (20–29, 30–39, 40–49, 50–59, 60–69, and 70–79 years); the mean RPKM value for each gene was calculated in different groups, respectively. STEM analysis (Ernst & Bar‐Joseph, 2006) method was adopted to analyze gene expression dynamic during muscle aging.

### 4.4 Enhancer analysis

Enhancer and super enhancer identification was performed following our previous method (Peng et al., 2017). Briefly, the H3K27ac‐enriched regions with a distance larger than 5 kb from the nearest TSSs were identified as enhancers. ROSE (Rank Ordering of Super‐Enhancers) algorithm (https://bitbucket.org/young_computation/rose) was then used to identify SEs (Hnisz et al., 2013; Whyte et al., 2013). Similarly, STEM analysis (Ernst & Bar‐Joseph, 2006) was applied to identify the enhancer constitutes with a continuous increase or decrease in H3K27ac signal during muscle aging; the nearest adjacent gene was assigned to an enhancer constitute as its target gene.

### 4.5 Animals, experimental design

C57/BL6 mice were housed in groups of five and maintained under controlled temperature (20 ± 1°C), humidity (55 ± 10%), and illumination (12/12 hr light cycle with lights on at 07:30 a.m.). Food and water were provided ad libitum. All mice were held in quarantine for 2 weeks before the experiments. Tubes for tunneling and nesting materials (paper towels) were routinely placed in all cages as environmental enrichment. All procedures involving animal care or treatments were approved by the Animal Ethics Committee at Chinese University of Hong Kong (CUHK) on the protection of animals used for scientific purposes. Briefly, all mice were housed in the above condition for 2, 10–12, and 20 months; and then, H3K27ac ChIP‐seq was performed on TA muscle tissues from the mice. To investigate the JQ1 treatment effect on ECM gene activation, intraperitoneal injection of JQ1 at 50 mg/kg daily was performed on 10‐month‐old mouse for 14 days (Segatto et al., 2017). Then, TA muscle tissues were extracted from mice with JQ1 and control DMSO‐treated mice. Meanwhile, SCs were isolated from mouse muscle by using fluorescence‐activated cell sorting (FACS) with cell surface marker Sca1^−^/CD31^−^/CD45^−^/Vcam^+^ (Liu, Cheung, Charville, & Rando, 2015; Chen et al. 2019). The BD FACSAria Fusion cell sorter (BD Biosciences) was used for muscle SC sorting following the manufacturer's instructions.

### 4.6 Western blotting

Western blot analysis was performed following the standard procedures. Briefly, total proteins from muscle were lysed in RIPA lysis buffer supplemented with protease inhibitor cocktail and mechanically sheared using a homogenizer with 5–6 strokes on ice. The protein concentration was determined using a Bradford protein assay kit (Bio‐Rad). The following antibodies and dilutions were used: Histone H3‐K27 acetylation (Abcam, ab4729, rabbit polyclonal, 1:5,000); Brd4 (A301‐985A100, Bethyl Laboratories, 1:1,000); Timp2 (Santa Cruz, sc‐21735, 1:500); and α‐Tubulin (Santa Cruz, sc‐23948, 1:5,000). Quantification of the western bands was performed using Image J software.

### 4.7 Immunofluorescence and H&E staining

Immunofluorescence (IF) on cells was performed using the following antibodies: MyHC (Developmental Studies Hybridoma Bank, 1:50), Myogenin (Santa Cruz Biotechnology; 1:350), and ERTR7 (Hycult Biotech, HM1086; 1:20). Frozen muscle sections were prepared by immersion in OCT in liquid nitrogen. H&E staining on frozen muscle sections was performed according to standard procedures.

### 4.8 ChIP and ChIP‐seq

ChIP assays were performed as previously described (Peng et al., 2017; Zhou et al., 2015). For ChIP, TA muscle tissues were homogenized on ice and cross‐linked with 1% formaldehyde at room temperature for 10 min; cross‐linking reaction was quenched by addition of 0.125 M glycine for 10 min. Chromatin was fragmented using sonicator (S220, Covaris), followed by incubation with 5 μg of antibodies at 4°C for overnight. Antibodies for histone H3‐K27 acetylation (Abcam, ab4729, rabbit polyclonal) and Brd4 (A301‐985A100, Bethyl Laboratories) were used for ChIP, or normal rabbit IgG (Santa Cruz Biotechnology, sc‐2027) was used as negative control. Immunoprecipitated genomic DNA was resuspended in 50 μl of water. PCRs were performed with 1 μl of immunoprecipitated DNA as template with SYBR Green Master Mix (Life Technologies), and products were analyzed by qRT‐PCR on a 7900HT system (Life Technologies). Primers used are listed in Table S5.

### 4.9 qRT‐PCR

Total RNA from cells and muscle tissues were extracted using TRIzol reagent (Life Technologies) according to the manufacturer's instructions, and cDNAs were prepared using PrimeScript™ RT Master Mix kit (Takara, RR036A). Analysis of mRNA expression was performed with SYBR Green Master Mix (Life Technologies) as described on a 7900HT System (Life Technologies). All primers are listed in Table S5.

## CONFLICT OF INTEREST

None declared.

## AUTHOR CONTRIBUTIONS

Conceived and designed the experiments: H.W. and J.Z. Performed the experiments: K.K.S., Y.L., Y.L., J.L., W.L., Z.J., and F.C. Analyzed the data: J.Z., J.Y., Y.D., Y.H., and G.L. Wrote the paper: H.W., J.Z., and K.K.S. Reviewed and edited the manuscript: H.W., J.Z., K.K.S, Y.L., and H.S.
