# Supplementary material for: Elevated H3K27ac in aged skeletal muscle leads to increase in extracellular matrix and fibrogenic conversion of muscle satellite cells
Source: Aging Cell. 2019 Jul 20;18(5):e12996. doi: 10.1111/acel.12996 (PMC6718601; doi:10.1111/acel.12996)
Supplement: Supplementary file 1 [file ACEL-18-e12996-s001.pdf]

Figure S1. Zhou J, So K and Li Y et. al.

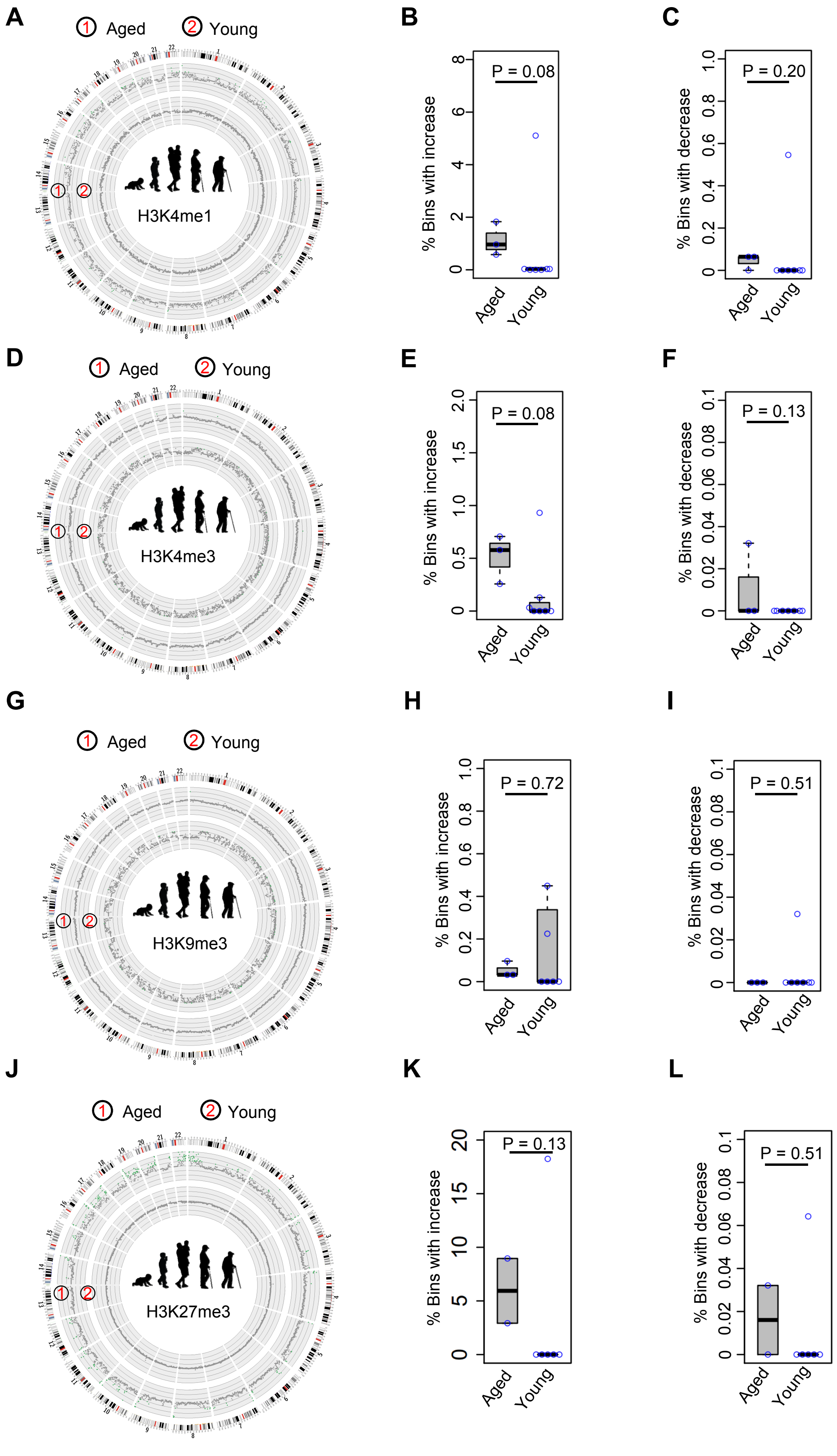

Figure S1. Zhou J, So K and Li Y et. al. (Continued)

M

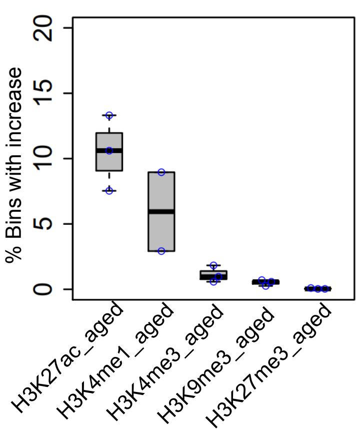

N

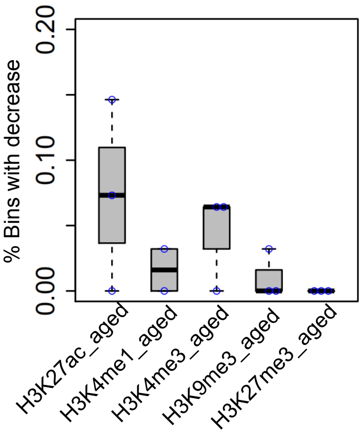

Figure S1. Comparison of a panel of histone modifications between young and aged human muscle tissues. (A-L) Comparison of H3K4me1 (A-C), H3K4me3 (D-F), H3K9me3 (G-I) and H3K27me3 (J-L) between young and aged human muscle tissues. (M) Boxplot showing that the percentage of bins with increased histone modifications in the aged group. (N) Boxplot showing that the percentage of bins with decreased histone modifications in the aged group.

Figure S2. Zhou J, So K and Li Y et. al.

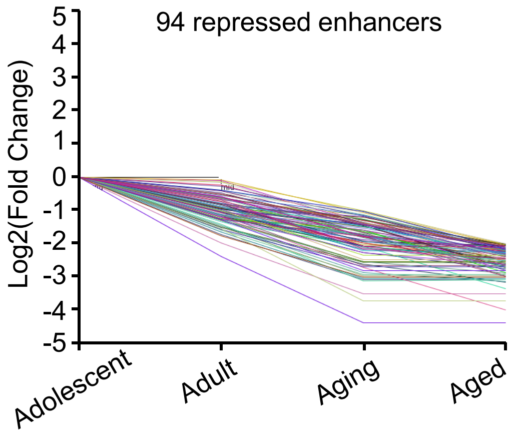

Figure S2. Line plot showing the fold change (log2) of enhancer constituents with decreased H3K27ac signal during muscle aging. The enhancer cluster is identified through STEM analysis.

Figure S3. Zhou J, So K and Li Y et. al.

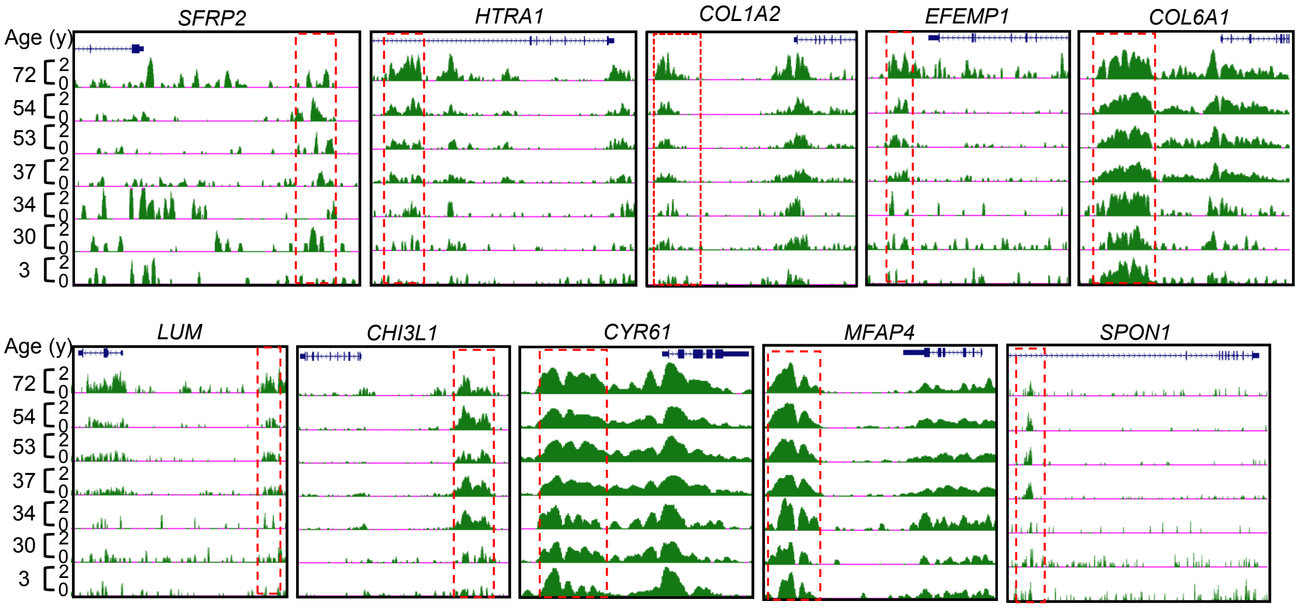

Figure S3. Snapshots of H3K27ac signals in cognate enhancers of ECM genes shown in Figure 3G.

Figure S4. Zhou J, So K and Li Y et. al.

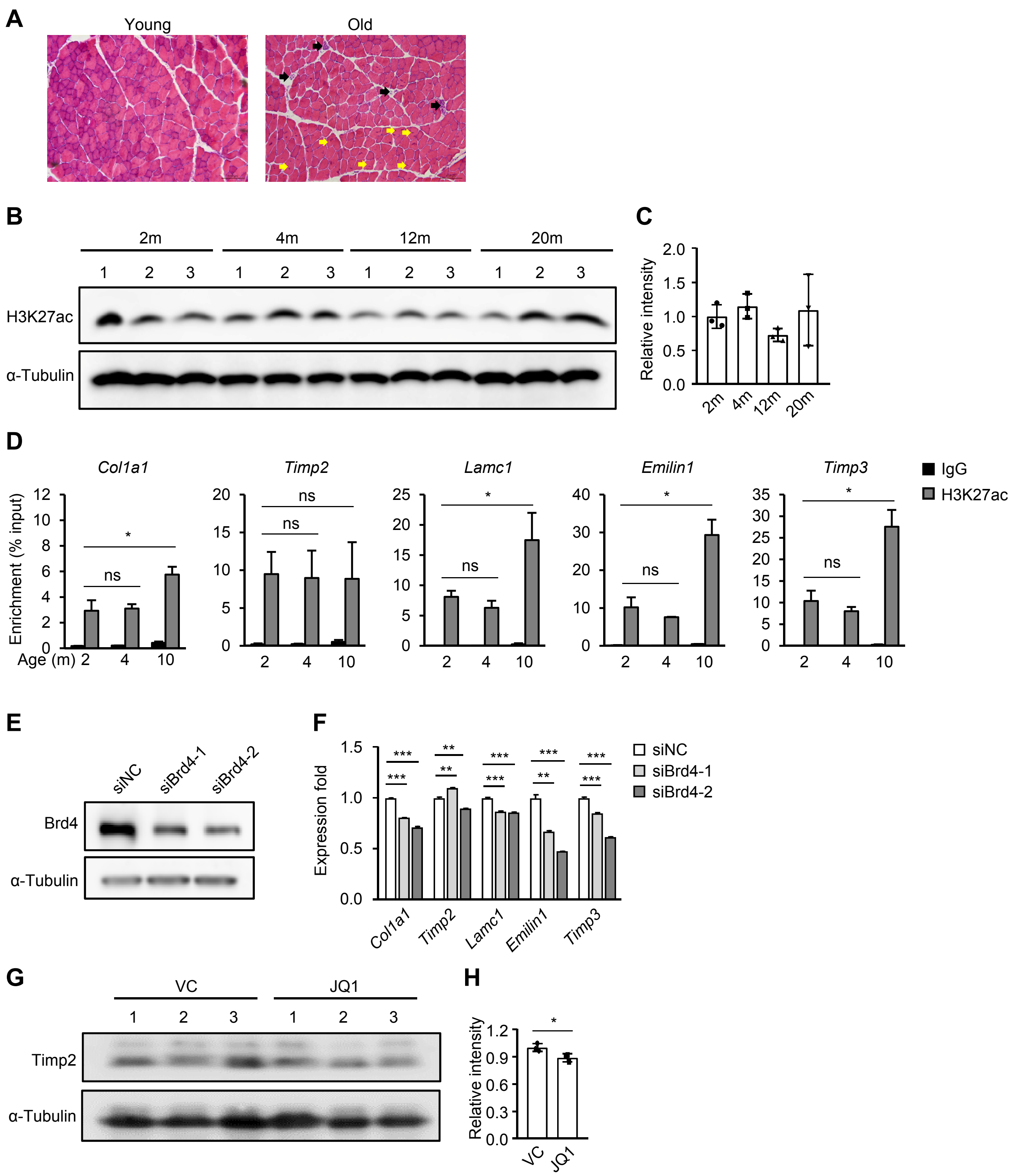

Figure S4. JQ1 treatment reverts the expression of some ECM genes in aged muscle. (A) H&E staining of cross-sections of TA muscles from young (2-month) or aged mice (20-month). Black arrows indicate degenerated myofibers; yellow arrows indicate regenerated myofibers. (B) Total H3K27ac protein levels in muscles of 3 mice at the indicated ages were quantified by Western blot and (C) normalized to α-Tubulin. n = 3 mice per age group. (D) Binding of H3K27ac on selected ECM enhancers by ChIP-PCR. (E) Two different siRNA oligos against Brd4 were transfected into C3H/10T1/2 fibroblasts. At 48h post transfection, the expression of Brd4 protein was decreased as measured by Western blot. (F) The mRNA levels of the indicated ECM genes in the above cells were measured by RT-qPCR. (G) Protein level of Timp2 in skeletal muscle treated with VC or JQ1. (H) Timp2 was normalized to α-Tubulin in (G) and decreased in skeletal muscle treated with JQ1. n = 3 mice per group.

Figure S5. Zhou J, So K and Li Y et. al.

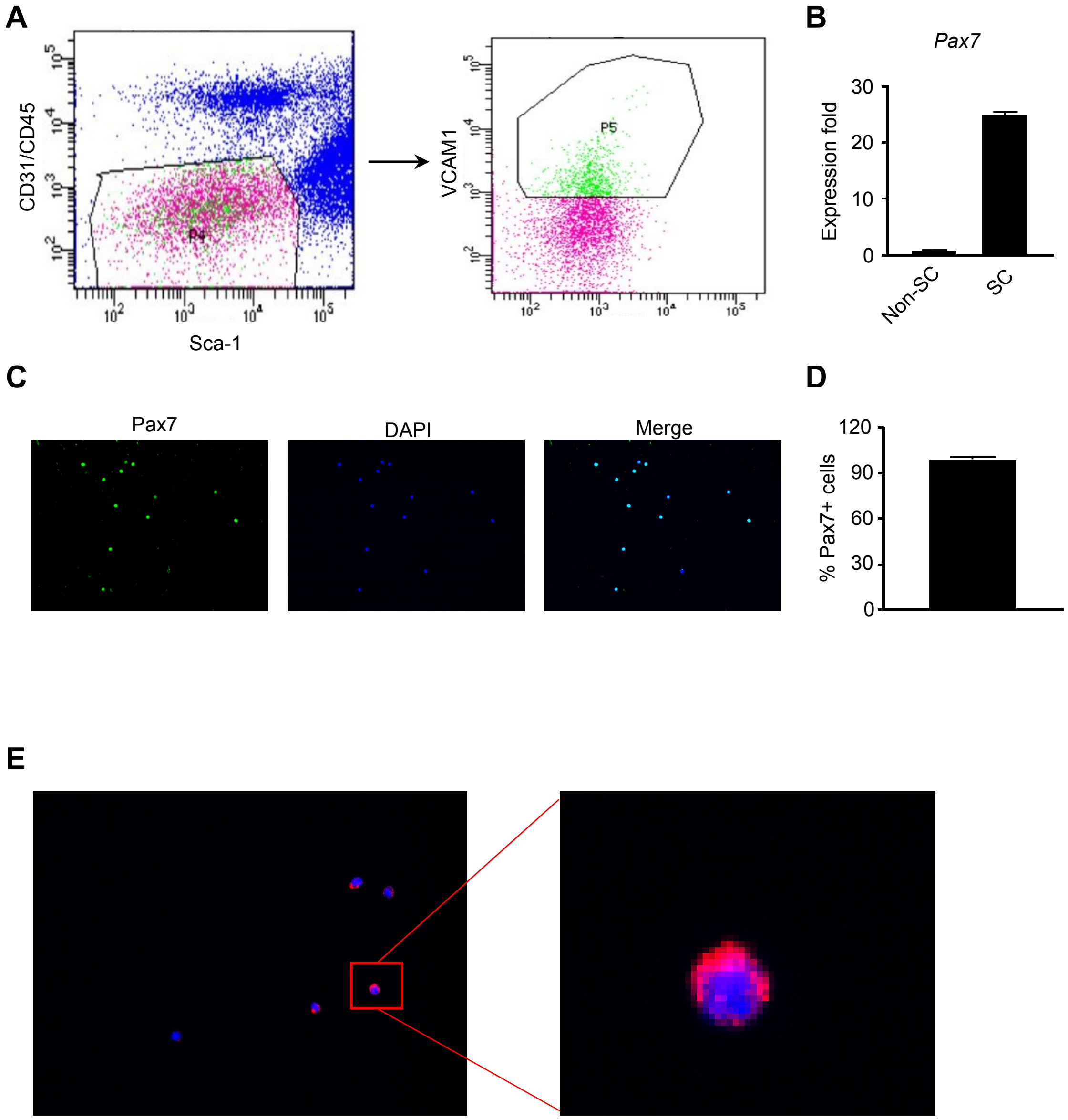

Figure S5. High purification of satellite cells were isolated from mouse skeletal muscles by fluorescence-activated cell sorting (FACS). (A) SCs were sorted from limb muscle of mouse using CD31-/CD45-/Sca-1-/VCAM+ profiles. (B) RT-qPCR analysis of Pax7 mRNA levels in the above fresh isolated SC and non SC cell populations. (C) Immunofluorescence (IF) staining of Pax7 in the above SCs. (D) The percentage of Pax7+ cells is shown. (E) IF staining of SCs freshly isolated from aged mouse (10month) for ERTR7 (red); high-magnification image of the boxed region is shown on the right.
